# P-2105. Concordance of Multiplexed Molecular Lower Respiratory Panel with Standard Culture Method and Potential Clinical Impact on Antimicrobial Therapy

**DOI:** 10.1093/ofid/ofae631.2261

**Published:** 2025-01-29

**Authors:** Nisha M Patel, Melissa R Gitman

**Affiliations:** NYU Grossman School of Medicine, New York, New York; Icahn School of Medicine at Mount Sinai, New York, NY

## Abstract

**Background:**

Pneumonia is the most common cause of health-care associated infections. The mainstay of diagnosis is respiratory culture, which is limited by up to 72-hour turnaround time and variable sensitivity. An added challenge is the emergence of antimicrobial resistance, which may confound the choice for empirical therapy. Rapid diagnostic tests have been developed to mitigate the challenges of diagnosis and treatment, but the clinical impact is not known. The Unyvero Lower Respiratory Tract Panel (LRT) is a qualitative multiplex nucleic acid test that can detect DNA of 20 microorganisms and 10 resistance genes in respiratory samples, with a 5-hour turnaround time. We hypothesized that use of the LRT on respiratory samples may have an impact on antimicrobial therapy and, consequently, antimicrobial stewardship.

**Methods:**

The LRT was performed on a random sample of 48 respiratory tract specimens that were obtained inpatient. We assessed concordance of LRT with standard-of-care (SoC) testing. We performed a retrospective chart review for the clinical context in which specimens were obtained (infection suspected versus not) and to determine whether use of LRT would have had an impact on antimicrobial therapy at the time of care.

**Results:**

Of 48 specimens, 25 (52%) had concordance between LRT and SoC testing and the remaining 23 specimens (48%) had discordance between LRT and SoC testing. Notably, 8 (17%) “discordant” specimens had organisms grow on culture that have no targets on the LRT (*Candida* species, *Klebsiella aerogenes*, *Burkholderia cepacia* complex, *Streptococcus anginosus*). In 3 cases where a resistance gene (CTX-M, mecA) was identified by LRT, antimicrobial susceptibility testing was compatible. In 7 cases, antibiotic therapy may have been impacted by use of LRT; in 5 cases therapy may have been initiated unnecessarily, and in 2 cases additional therapy may have been warranted. There were no cases where de-escalation of antibiotics would have been warranted.

**Conclusion:**

The Unyvero LRT panel is an appealing diagnostic tool due to fast turnaround time, but a significant clinical impact due to its use has not yet been demonstrated. Though our study was limited by sample size, we found that LRT may have increased inappropriate use of antibiotics, rather than promoting stewardship.

**Disclosures:**

Melissa R. Gitman, MD, Biomerieux, Inc: Grant/Research Support

